# The public sector nursing workforce in Kenya: a county-level analysis

**DOI:** 10.1186/1478-4491-12-6

**Published:** 2014-01-27

**Authors:** Mabel Wakaba, Patrick Mbindyo, Jacob Ochieng, Rose Kiriinya, Jim Todd, Agnes Waudo, Abdisalan Noor, Chris Rakuom, Martha Rogers, Mike English

**Affiliations:** 1 Wellcome Trust Collaborative Programme, Kenya Medical Research Institute Centre for Geographic Medical Research Coast, PO Box 43640-00100 GPO, Nairobi, Kenya; 2 Emory University Kenya Health Workforce Project, PO Box 7808-00200, City Square, Nairobi, Kenya; 3 Department of Nursing, Ministry of Medical Services, PO Box 30016-00100, Nairobi, Kenya; 4 London School of Hygiene and Tropical Medicine, Keppel Street, London WC1E 7HT, UK; 5 Lillian Carter Center for Global Health and Social Responsibility, Emory University, 325 Swanton Way, Decatur, GA 30030, USA; 6 Nuffield Department of Medicine, John Radcliffe Hospital, Centre for Tropical Medicine, University of Oxford, Oxford, UK; 7 Department of Paediatrics, John Radcliffe Hospital, University of Oxford, Oxford, UK

**Keywords:** Nurses, Public sector, Human resources for health, County, Kenya

## Abstract

**Background:**

Kenya’s human resources for health shortage is well documented, yet in line with the new constitution, responsibility for health service delivery will be devolved to 47 new county administrations. This work describes the public sector nursing workforce likely to be inherited by the counties, and examines the relationships between nursing workforce density and key indicators.

**Methods:**

National nursing deployment data linked to nursing supply data were used and analyzed using statistical and geographical analysis software. Data on nurses deployed in national referral hospitals and on nurses deployed in non-public sector facilities were excluded from main analyses. The densities and characteristics of the public sector nurses across the counties were obtained and examined against an index of county remoteness, and the nursing densities were correlated with five key indicators.

**Results:**

Of the 16,371 nurses in the public non-tertiary sector, 76% are women and 53% are registered nurses, with 35% of the nurses aged 40 to 49 years. The nursing densities across counties range from 1.2 to 0.08 per 1,000 population. There are statistically significant associations of the nursing densities with a measure of health spending per capita (*P* value = 0.0028) and immunization rates (*P* value = 0.0018). A higher county remoteness index is associated with explaining lower female to male ratio of public sector nurses across counties (*P* value <0.0001).

**Conclusions:**

An overall shortage of nurses (range of 1.2 to 0.08 per 1,000) in the public sector countrywide is complicated by mal-distribution and varying workforce characteristics (for example, age profile) across counties. All stakeholders should support improvements in human resources information systems and help address personnel shortages and mal-distribution if equitable, quality health-care delivery in the counties is to be achieved.

## Background

The global shortage of health workers is estimated at more than four million, assuming that all countries attain an average worker density of 2.5 per 1,000 population (counting only doctors, nurses, and midwives) [1]. According to the World Health Organization’s World Health Report 2006, based on data in the Global Atlas of the Health Workforce for 193 member states, there are currently 57 countries including Kenya with critical shortages. These together have a deficit of 2.4 million doctors, nurses, and midwives, with the proportional shortfalls being greatest in sub-Saharan Africa [2]. Indeed sub-Saharan African countries must nearly triple their current number of workers if they are to achieve the Millennium Development Goals for health [1].

Studies commissioned by the Joint Learning Initiative examined the national patterns of worker density in relation to key variables, such as national income, child and maternal mortality, and expenditure in health care [1]. Most striking are the low-income countries, where low density of workers hinders their capacity to cope with the health crisis. Other studies in hospitals and nursing homes in high-income countries also demonstrated the importance of worker density and quality of health-care outcomes [3-10]. One cross-country econometric study showed that as the density of health workers increases, maternal, infant, and under-five mortality all decline [11]. Another study done using data in 49 countries found that the combined density of doctors and nurses to population is positively and significantly related to coverage of three vaccines when controlling for other health determinants [12]. The correlation between the availability of health workers and coverage of health interventions suggests that the public’s health suffers when health workers are scarce [11,13-16].

Nurses are the main professional component of the ‘front line’ staff in most health systems and their contribution is recognized as essential to delivering safe and effective care, with links demonstrated between adequate nurse staffing levels and positive care outcomes. However the low availability of nurses in many developing countries is exacerbated by geographical mal-distribution - there are even fewer nurses available in rural and remote areas [17]. Furthermore national averages of workforce density often hide marked inequalities in distribution, such as across geographical areas and employment sectors [18]. It has been found that the prerequisite for effective deployment of staff is an information system that enables management and nurses to review patterns of activity and variations in workload that can be used to inform staffing levels [17]. Health information systems that collect, analyze, report, and use up-to-date health information are necessary for generating, managing, and disseminating knowledge on the health workforce. Effective action, both urgent and sustained, requires solid information, reliable analyses, and a firm knowledge base [1].

Recognizing this, the Kenya Health Workforce Information System was developed through a collaborative initiative of the Ministries of Health - Department of Nursing, the Nursing Council of Kenya, the US Centers for Disease Control and Prevention, and the Lillian Carter Centre for Global Health and Social Responsibility at Emory University, Atlanta [19]. The project’s immediate objective was the creation of a computerized database system for Kenya’s nursing workforce with a long-term goal of providing accurate workforce data capable of informing national human resources for health policy and decision-making. The development of this information system has been described elsewhere [20]. In brief, however, data exist in two main forms. Supply side data on all nurses, now referred to as the Regulatory Human Resources Information System, are maintained by the Nursing Council of Kenya while deployment data for qualified staff predominantly working in the public sector are maintained by the Ministries of Health in the Kenya Health Workforce Information System.

The Kenya Health Policy, which guides attainment of the long-term health goals sought by the country, defines four main levels of care, that is, community, primary, county, and national. The public sector stewards of the health sector include the national ministry responsible for health, the county department responsible for health, and professional regulatory bodies [21]. Prior to Kenya’s new constitution taking effect after the March 2013 general elections, there were two health-related ministries, the Ministries of Medical Services and Public Health and Sanitation, now amalgamated in the Ministry of Health.

Previous reports based on analyses of these data have indicated variation in the coverage of nurses between Kenya’s former eight provinces [22]. Data have also been used to help evaluate the Emergency Hire Plan to support provision of essential services notably care and prevention of HIV/AIDS [19]. Since these analyses and following promulgation of a new constitution on 27 August 2010, radical changes to Kenya’s administrative systems are occurring with considerable devolution of powers to the country’s new counties.

As of the 2013 general elections in Kenya, there are 47 counties which are Kenya’s geographical units of devolved government. County boundaries largely reflect historical, political, and administrative units themselves largely influenced by ethnic, geographic, and political considerations. Based on Kenya’s new constitution, the health sector is being restructured such that the functions of the national government will include formulating health policy and managing national referral facilities, while the county governments’ functions will include facilitating provision of health services at county health facilities and managing their county workforce recruitment and retention. Health professional regulatory bodies will continue facilitating the registration and licensing of health workers nationally.

As Kenya transfers the responsibility for health-care delivery to county governments, it is critical to understand their capacity, in terms of the health workers they are likely to inherit, and to examine the distribution of this capacity. Furthermore, perhaps for the first time, the improved human resources information becoming available allows the relationship between nursing workforce density and key health indicators to be examined within a single low-income African country.

A widely used health workforce threshold is 2.5/1,000 for doctors, nurses, and midwives. Although concentrating on nurses alone in this work, we retain this threshold as a general reference point noting that in the Kenyan health sector there are about 10 nurses for each doctor in the public sector [23].

## Methods

De-identified deployment data comprising staff returns and staff details data from the Kenya Health Workforce Information System were provided as of 2012. The staff returns data spanning all nurses salaried by the public sector are based on periodic staff returns forwarded to the Ministries of Health from district and provincial administrations. Data on nurses deployed in non-public sectors (faith-based, non-governmental, and private) are rarely forwarded as they are not clearly mandated to do so.

All analyses therefore concern public sector nursing workforce provision in government health facilities and administrative institutions directly supporting health service delivery. Notably the Government of Kenya is the largest employer of the country’s health workforce with over 50% of health workers deployed in the public sector [23]. Furthermore it is estimated that nurses comprise 45.3% of the total public sector health workforce [24]. This nursing workforce includes long-term government employees and those employed under contract through projects funded by, for example, the Economic Stimulus Package. These nurses were included in the analysis because they are expected, eventually, to be absorbed into public service.

The private health sub-sector comprises of for-profit and not-for-profit health-care institutions. The latter include faith-based health institutions and non-governmental organizations providing health-care. The number of nurses in the private sector is lower than that in the public sub-sector but while the Ministries of Health have data on their recruited health staff, there are no centrally consolidated data for private health sector employees. This is because the private health sector is fragmented and diverse in ownership, structure, and operations [24], although this sector often employs doctors and nurses who are also found within the public health sector [25].

The staff details data from the public sector contains demographic particulars of the nurses such as gender. The nurses’ qualifications specifically were obtained from the Regulatory Human Resources Information System since the system contains information on training, registration, and licensing of nurses. Generally nurses in Kenya are broadly categorized by qualification into enrolled and registered nurses. Primary training lasts 2.5 years for the enrolled nurses at certificate level, and registered nurses undergo either a 3.5-year diploma course or a 4-year degree (BScN) programme. Both the registered and enrolled nurses are of varying cadres. In practice, registered nurses provide both general and specialized care and play managerial functions [24]. Enrolled nurses are entry-level nurses that work under the supervision of registered nurses [26]. However, various health-care tasks can be carried out by the different nursing cadres, for example, giving immunizations. Local training institutions are the main source of Kenya’s supply of nurses, as foreign supply is negligible [24]. As at the end of 2012 there were about 83 active nurse training institutions and over 4,000 nurses graduating annually.

**Figure 1:**
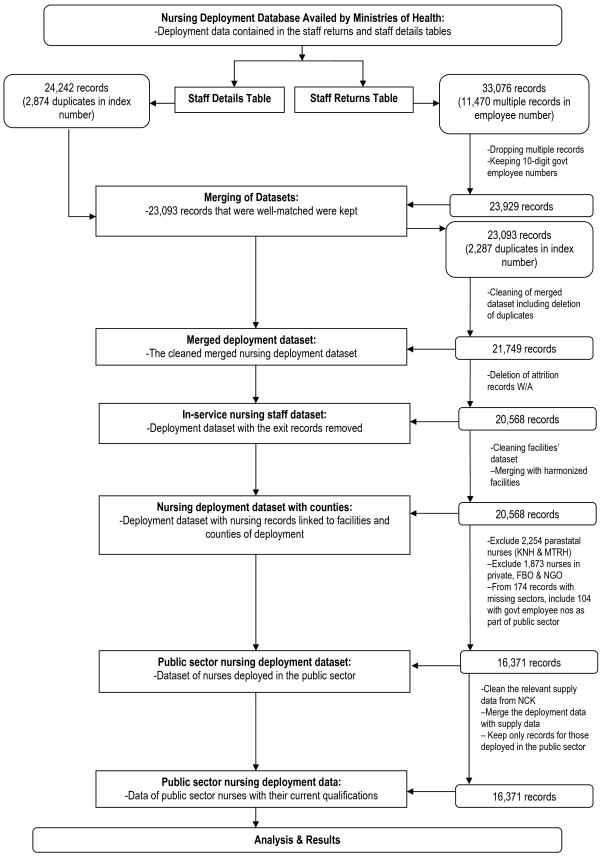
**Methods used to obtain the public sector nursing staff data.** Illustrated are the methods used to clean the data from the Ministries of Health, to extract the data on the 16,371 nurses deployed in the public sector (excluding those in national referral hospitals) and merging the same to data from the Nursing Council of Kenya so as to obtain the qualifications of the public sector nurses.

The deployment data were taken through an iterative cleaning process including matching nursing records from the staff returns and staff details data that are described above, elimination of duplicates, and also nursing attrition records were removed so as to provide the best available representation of the current in-service nursing workforce. The facilities to which the nurses were deployed had assigned standard geographical codes (master facility list codes) within counties, and where these were missing, available district and province codes were used to allocate the nurses to counties of deployment where possible. The exclusions of data on nurses deployed in the non-public sectors and in national referral hospitals were then made, and the data were then linked to the current qualifications of the nurses. The resulting deployment data used for analysis had 16,371 public sector nursing records (Figure 1).

National referral hospitals (that is, Kenyatta National Hospital and Moi Teaching and Referral Hospital) provide highly specialized health care, facilitate health-related research and training, and they deal with referrals from across the country. County hospitals should support a network of primary care facilities so that together they ensure delivery of a comprehensive essential package of care. Hence referral hospitals were excluded from analysis of the nursing workforce available to counties because they are a national resource and, due to their specialized nature, these hospitals will remain under the national Ministry of Health rather than being handed over to county administrations. Analyses that allocate their nursing complement to one county would furthermore distort the nursing workforce density in that county, unfairly suggesting it had a higher county-specific number of staff.

Statistical (STATA® version 11) and geographical (ARC-GIS® version 10) software were used for analysis. We examined the public sector nursing workforce distribution and characteristics against county remoteness levels (Panel 1) using linear regression, and also explored the relationship between nursing densities and key indicators (Panel 2) using correlation analysis. Scatter plots with fitted trend lines provided a visual representation of the linear association between the nursing densities and the county indicators.

Prior to correlation analysis, the data variables were examined for normality using graphical methods (plotting the variables’ histograms with overlaid normal density curves, stem and leaf plots, probability-probability plots, and quantile-quantile plots), and statistical methods (skewness and kurtosis, Shapiro-Wilk W and Shapiro-Francia W’ tests). Some variables exhibited non-normality and hence Spearman’s correlation analysis was used.

### Panel 1: Description of county remoteness index

Generalized indices of remoteness are useful for service planning and equitable distribution of resources and also for assessing health and workforce needs and the resources allocated to meet these needs. The remoteness index as used in this study is an index of accessibility to service centers.

To generate the remoteness index, population settlements in Kenya were classified by distance to three types of service centers, that is, service centers 1 (market and trading centers), service centers 2 (division headquarters and small towns), and service centers 3 (cities, municipalities, major towns, and provincial and district headquarters). The classification of service centers used in this study was developed by the Kenya National Bureau of Statistics for census purposes and was on the basis of population size and service functions. A surface of travel times at 1 × 1 km spatial resolution to various service center types was generated. Travel speeds were assigned to different land cover and land use classes, roads, and slope by assuming multiple modes of transport within a single journey to a service center.

From each grid pixel, the travel time to any category of service center was divided by the average travel time to that category. The result was a surface of ratio-to-mean travel time. For example, if a grid pixel had a ratio-to-mean travel time of 2 to service center 1, this implied that it took twice as long to reach the nearest service center 1 compared to other service centers. This ratio for each pixel was capped at a value of 0.5, equivalent to approximately 30 minutes to a service center 1; 1.5 hours to a service center 2, and 2 hours to a service center 3. All pixels where the ratio-to-mean to any service center was ≥0.5 were assigned a ratio-to-mean of 0.5. This was done to reduce the influence of the longer travel times to larger but fewer service centers on the overall index. The capped ratio-to-mean surfaces to each type of service center were summed, resulting in a continuous index of remoteness ranging from 0 to 1.5. For each county, the mean value was extracted, which was then classified into five categories as follows: highly accessible (0 - ≤0.3), accessible (>0.3 - ≤0.6), moderately accessible (>0.6 - ≤0.9), remote (>0.9 - ≤1.2), and very remote (>1.2 - 1.5). Further details about the methodology used for defining remote areas are described in Noor et al. [27].

### Panel 2: Description of selected county indicators

#### Urbanization

County estimates of the proportion of the population in an urban environment based on the classification employed in the census of 2009 [28]. Urban areas were defined as towns having a population of at least 2,000 inhabitants, connected to the main road network, and served as a market center for several smaller trading centers [29].

#### Delivery care provided by a skilled provider

County estimates of the proportion of children aged 0 to 59 months whose mothers had received care from a skilled birth attendant as based on the Kenya Integrated Household Budget Survey 2005/2006 data [30].

#### Health spending per capita

County estimates of spending on health per capita (Kenya Shillings) based on bed nets and illness, that is, the proportion of population that slept under a bed net by region, and the proportion of population that had a fever or malaria by region respectively, according to data from the Kenya Integrated Household Budget Survey 2005/2006 data [31].

#### Immunization rates

County estimates of the proportion of children aged 12 to 23 months that were immunized as based on the Kenya Integrated Household Budget Survey 2005/2006 data [32]. Vaccinations considered were Bacille-Calmette-Guerin (BCG), polio (1-3), diphtheria-pertussis-tetanus hepatitis B-Hib (DPT 1-3), and measles.

#### Poverty rates

County estimates of the proportion of the population living in poverty based on and as derived from the Kenya Integrated Household Budget Survey (KIHBS) data for constituencies in 2005/2006 [33]. The poverty lines were derived from the KIHBS data using the Cost-of-Basic-Needs (CBN) method, which stipulates a consumption bundle deemed to be adequate for ‘basic consumption needs’, and then estimates what this bundle costs in reference prices. Consumption data used were adjusted for differences in needs based on household consumption (using adult equivalence scales that are used to convert household consumption aggregates into money metric measures of individual welfare), and nominal food expenditure were adjusted for spatial and temporal price differences (given that prices in Kenya vary geographically and by season, an index was constructed that simultaneously adjusted for cost-of-living differences over both space and time) [34].

## Results

The data show that there are 18,625 nurses deployed in the public sector including 2,254 (12%) nurses within two national referral hospitals. Of the 16,371 nurses deployed in the public, non-tertiary sector that are the main focus of this study, 12,474 nurses (76%) are women and 8,741 nurses (53%) are registered nurses. More specifically the community health nursing cadres (basic courses) have the highest nurse numbers, that is, Kenya Registered Community Health nurses and Kenya Enrolled Community Health nurses comprise 45% and 34% of public sector nurses respectively (ratio 1.3:1). Community health nurses mainly work in the community setting as public health nurses, and they are usually skilled in general nursing, midwifery, and community nursing.

**Figure 2:**
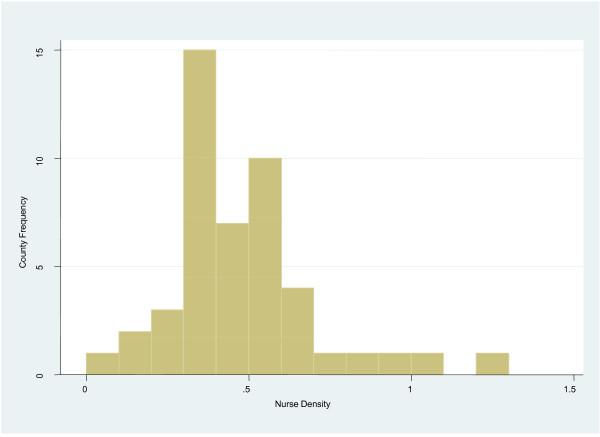
**Public sector nurse density by county frequency.** The frequencies of the public sector nurse to population densities across the counties (*n* = 47) are shown. The vertical axis of the histogram represents the county frequency while the horizontal axis represents the public sector nursing densities per 1,000 population.

**Figure 3:**
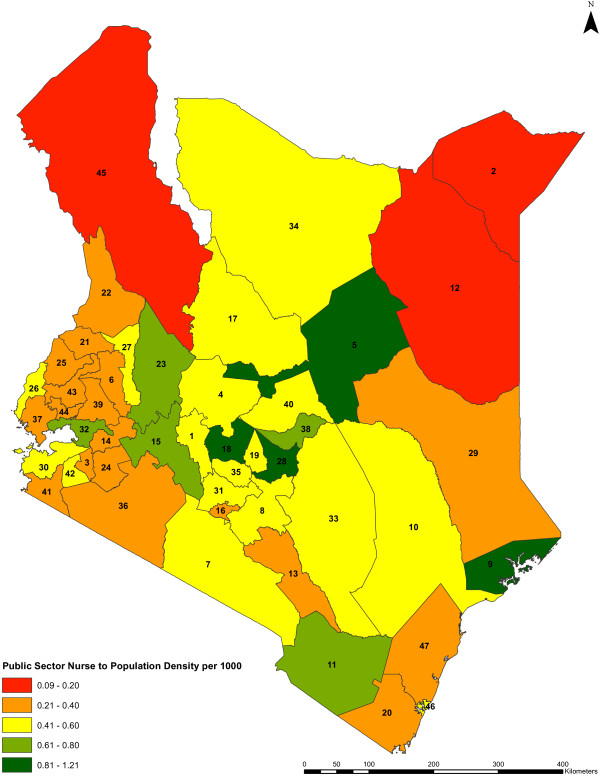
**Public sector nurse to population density by county.** The public sector nursing densities per 1,000 population across the counties (*n* = 47) are shown. The colors indicate different range of values of the nursing densities for the counties, while the counties are represented by county identification numbers ranging from 1 to 47.

The national public sector nurse to population density is 0.42 per 1,000 and 0.48 per 1,000, excluding and including tertiary level nurses, respectively. The public sector nursing densities across counties vary from 1.2 per 1,000 to a low of 0.08 per 1,000, a 15-fold variation (Figures 2 and 3). In the two counties that host national referral hospitals, nursing densities are 0.30 per 1,000 and 0.26 per 1,000 if the nurses deployed in these hospitals are excluded, but rise to 0.82 per 1,000 and 0.96 per 1,000, respectively, if these nurses are included.

### Nurses’ gender, ages, and qualifications

**Figure 4:**
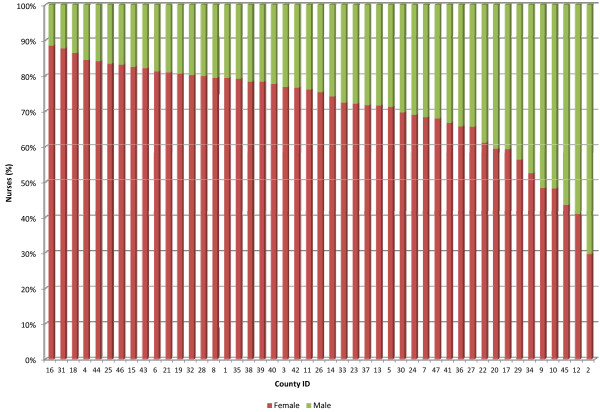
**Gender distribution of public sector nurses by county.** The proportions of female to male nurses that are deployed in the public sector across the counties (*n* = 47) are shown. The percentage of the public sector male nurses in each county is shown in green while the percentage of the public sector female nurses is shown in red. The counties are represented by county identification numbers ranging from 1 to 47.

**Figure 5:**
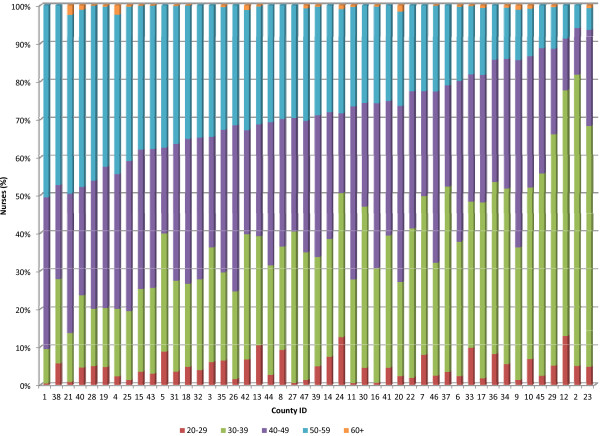
**Age distribution of public sector nurses by county.** The proportions of the public sector nurses based on their ages are categorized into five age groups across the counties (*n* = 47). The age groups are mainly classified into 10-year age bands. The percentage of the public sector nurses aged 20 to 29 years in each county is shown in red, the percentage of nurses aged 30 to 39 years is shown in green, the percentage of nurses aged 40 to 49 years is shown in purple, the percentage of nurses aged 50 to 59 years is shown in blue, and the percentage of public sector nurses aged 60 years and above is shown in orange. The counties are represented by county identification numbers ranging from 1 to 47.

**Figure 6:**
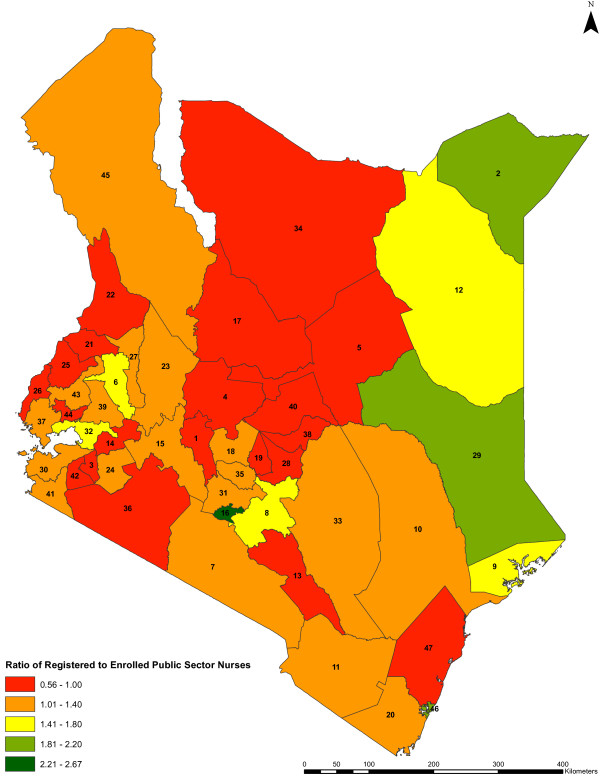
**Ratio of registered to enrolled public sector nurses by county.** The ratios of registered nurses to enrolled nurses in the public sector across the counties (*n* = 47) are shown. The colors indicate the different range of values of the qualification ratios in the counties, while the counties are represented by county identification numbers ranging from 1 to 47.

In 13 of the 47 counties over 80% of public sector nursing staff are women (Figure 4). The largest group of nurses (35%) are aged 40 to 49 years. The average age of this nursing workforce is approximately 44 years. Forty-six counties have over 50% of their public sector nursing workforce aged below 50 years, but in 20 counties over 30% of these nurses are aged 50 years and above (Figure 5). Only 4% of the nurses are aged below 30 years. Also the public sector registered nurse to enrolled nurse ratios range from 0.56 to 2.67 across counties (Figure 6). Of public sector nurses, 91% have midwifery skills.

### Tertiary level nurses

Of the 2,254 nurses deployed in the referral hospitals, 1,847 (82%) nurses are women and 80% are aged below 50 years with the largest group (43%) being aged 30 to 39 years. Likely reflecting their specialist status the ratio of registered to enrolled nurses in these hospitals combined is 3.41:1. When including these nurses in the deployment data for the two counties in which these centers are based, the nursing qualification ratios (registered to enrolled) change from 2.67 and 1.64 to 2.83 and 3.65, respectively.

### Cross-county analysis

**Figure 7:**
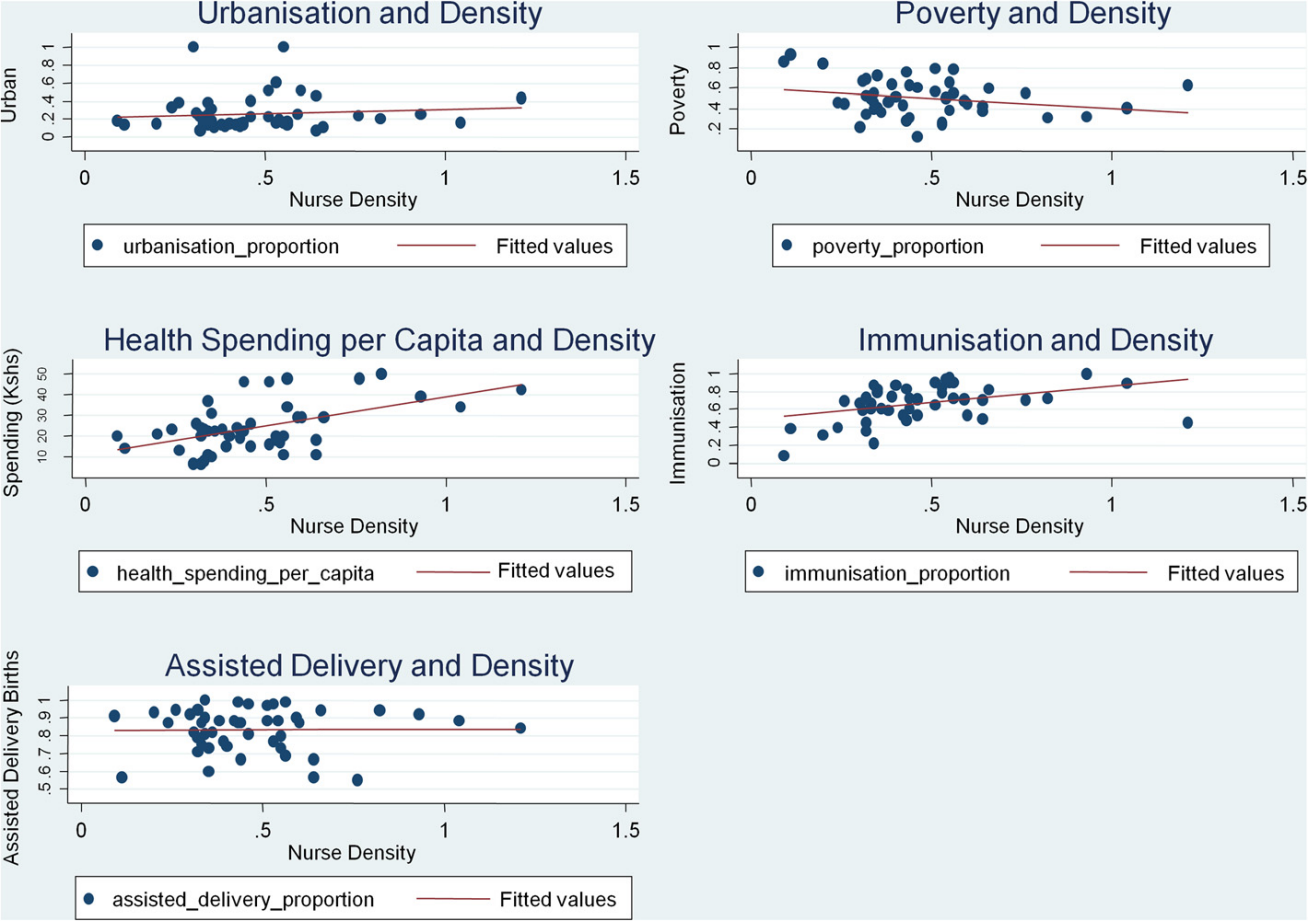
**Public sector nursing densities against selected county indicators.** The scatter plots of five selected county indicators (urbanization, poverty rates, health spending per capita, immunization rates, and delivery care provided by a skilled provider) plotted against the public sector nursing densities across the counties (*n* = 47) are shown, with corresponding fitted trend lines.

Results indicate that the public sector nursing densities (excluding tertiary level nurses) are positively associated with urbanization, health spending per capita, and immunization rates, and negatively associated with poverty rates and delivery care provided by a skilled provider (Table 1; Figure 7). From among the available county indicators correlation coefficients imply statistically significant associations of the county specific nursing densities with health spending per capita and immunization rates. On including tertiary level nurses, statistically significant associations are found between nursing densities and urbanization, poverty rates and immunization rates, yet no significant association is found between nursing densities and delivery provided by a skilled provider (although the association becomes positive) (Table 1).

**Table 1:** Correlation analysis between public sector nurse densities and selected county indicators

**Indicator against nurse density**	**r**_**s**_^**a **^**(*P *** **value)**	**r**_**s**_^**a **^**(*P *** **value)**
**(*n*** **= 47)**	**(excluding tertiary level nurses)**	**(including tertiary level nurses)**
Urbanization	0.1321 (0.3760)	0.3110 (0.0334)
Poverty rate	−0.2133 (0.1500)	−0.3250 (0.0258)
Health spending per capita	0.4263 (0.0028)	^b^
Immunization rates	0.4444 (0.0018)	0.4120 (0.0040)
Delivery care provided by a skilled provider	−0.0224 (0.8812)	0.0988 (0.5086)

^a^r_s_ – Spearman’s correlation coefficient.

^b^Excluded since the tertiary national hospitals mainly utilize central funding separate from the national government hence their corresponding expenditure is not captured in the county level health spending per capita indicator.

The table shows the spearman’s correlation coefficients based on correlation analysis of five selected indicators and the public sector nursing densities (both with and without nurses deployed in tertiary centers) across the 47 counties, as well as the associated *P* values for the correlation coefficients.

**Figure 8:**
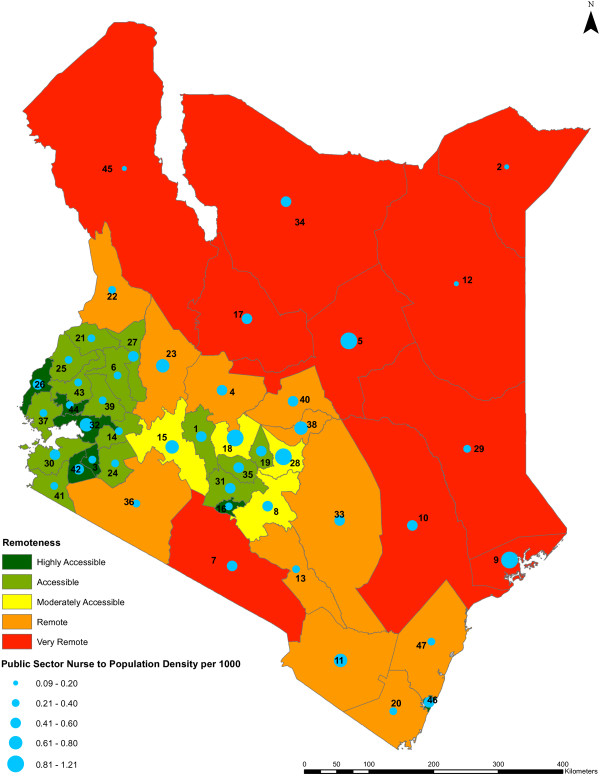
**Public sector nurse to population density against remoteness level by county.** The public sector nursing densities per 1,000 population across the counties against the remoteness levels of the counties (*n* = 47) are shown. The colors indicate different range of values representing the remoteness levels of the counties, from highly accessible counties being shown in dark green to very remote counties being shown in red. The nursing densities are represented by blue circles of different sizes which are based on the different range of values of the densities, while the counties are represented by county identification numbers ranging from 1 to 47.

**Figure 9:**
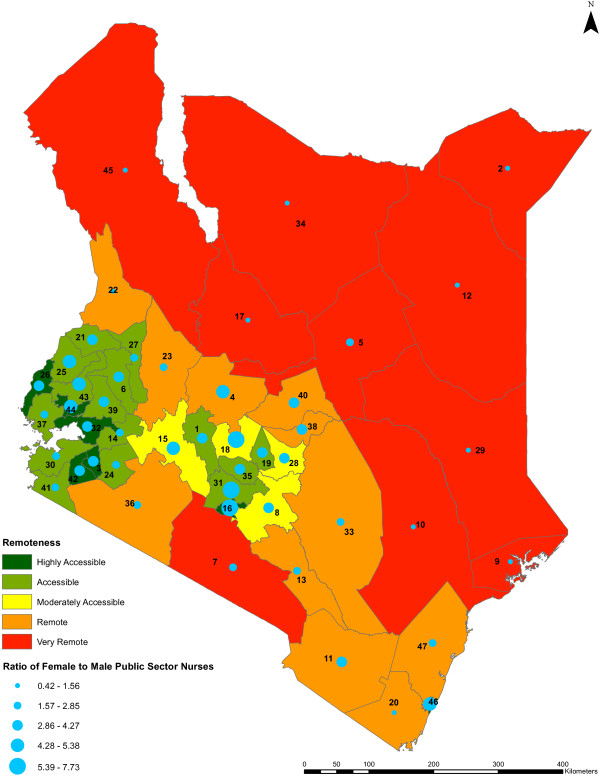
**Ratio of female to male public sector nurses against remoteness level by county.** The gender ratios of female nurses to male nurses in the public sector across the counties against the remoteness levels of the counties (*n* = 47) are shown. The colors indicate the value ranges representing the remoteness levels of the counties, from highly accessible counties being shown in dark green to very remote counties being shown in red. The gender ratios are represented by blue circles of different sizes which are based on the value ranges of the ratios, while the counties are represented by county identification numbers ranging from 1 to 47.

**Figure 10:**
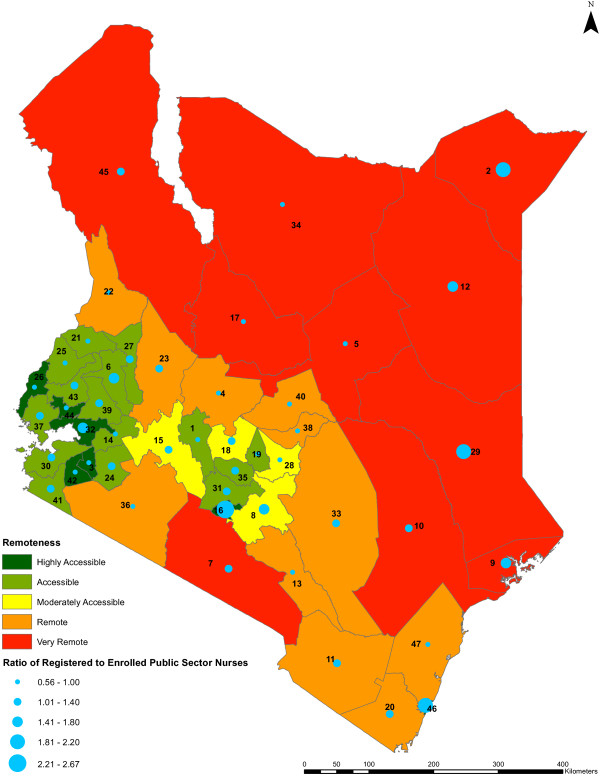
**Ratio of registered to enrolled public sector nurses against remoteness level by county.** The qualification ratio of registered nurses to enrolled nurses in the public sector across the counties against the remoteness levels of the counties (*n* = 47). The colors indicate different range of values representing the remoteness levels of the counties, from highly accessible counties being shown in dark green to very remote counties being shown in red. The qualification ratios are represented by blue circles of different sizes which are based on the different range of values of the ratios, while the counties are represented by county identification numbers ranging from 1 to 47.

We found no statistically significant relationship between nursing densities and county remoteness levels (*P* value = 0.5179) (Figure 8). Remoteness is associated with the female to male ratio of public sector nurses across the counties (*P* value <0.0001) but not with the registered to enrolled nurse ratios of the nurses (*P* value = 0.9129) (Figures 9 and 10, respectively).

## Discussion

The study results indicate that there is likely an overall shortage of nurses countrywide. Although we cannot include data from the private health sector this and inclusion of the much smaller number of doctors is unlikely to make up the apparently large gap between actual workforce densities and the minimum health workforce density recommended by the World Health Organization [2]. Of particular note, however, is that the data indicate a potential 15-fold variation in the nurse to population ratios across counties within the country. As Kenya devolves health-care delivery to these counties such mal-distribution is a major concern.

Interestingly the assessment of the variation of nursing density with three broad metrics of vulnerability, that is, poverty rates, remoteness, and urbanization across the counties showed no statistically significant associations (though significant associations of nursing density with urbanization and poverty rates become evident on including the tertiary level nurses). Possible reasons for this could be that either the county aggregation of data may be masking some important within county disparities and may require a finer unit of analysis, or that there is truly no general relationship. The latter perhaps reflects the recent efforts by government, with support from partners, to target recruitment to some hardship areas. Thus, a number of remote counties lying on Kenya’s borders with Somalia, Sudan, and Ethiopia still have a very low density of nurses as well as the highest poverty rates (Figure 8).

County level nurse densities should also be interpreted cautiously. In very large counties (that are often remote), nurse density, as a metric of access, might be better considered as a product of nurses per unit population and per unit area. The fact that service users may clearly not respect administrative boundaries should also suggest caution in interpreting absolute nurse density values. Intuitively in geographically very large counties those seeking health care are also least able to cross administrative borders in search of services to ameliorate the impact of local low nurse density. Finally it is clear that significant heterogeneity in access to services exists at much more local levels [35-37] and future work should aim to explore access in more detail. Nonetheless the analyses presented are useful at a political and management level as devolution progresses and clearly illustrate the value of developing improved national human resources for health information systems.

As with nurse to population densities, there appears to be inequity in the distribution of more highly qualified nurses in the public sector across counties. A five-fold difference in the highest and lowest ratios of registered nurses to enrolled nurses across the counties was found. Interestingly, a county’s remoteness level was associated with the gender balance of the workforce (a higher proportion of male nurses in remote counties) but not with level of qualification. This likely reflects a specific but informal policy of deploying male nurses to remote, hardship areas where possible. Gender analysis is important where potential issues such as cultural sensitivities among communities may arise, and which may have implications for workforce planning such as gender-related retention strategies.

There is an alarmingly low proportion of public sector nurses aged below 30 years. This is consistent with a case study conducted on nursing human resources in Kenya that indicated that the nursing population in the Ministries of Health is aging [24]. This could be due to the public sector hiring freeze that took place between 1994 and 2002. This resulted in a shrinking health workforce and a substantial pool of qualified health professionals especially nurses who were unemployed and available on the local labor market [38]. Despite the hiring freeze nursing education continued but a recent study reflects a strong supply of nursing graduates and the inadequate employment opportunities in Kenya [39]. Out-migration of nurses also contributes to the health workforce crisis in Kenya. A previous study has shown that for every 4.5 nurses Kenya adds to the nursing workforce through training, one nurse in the workforce applies to out-migrate, with 70% of nurses that applied to out-migrate being between the ages of 21 to 40 years [40].

Thus a number of counties have a relatively high proportion of nurses aged 50 years and above. These counties will inherit an aging nursing workforce, many of whom will soon reach the civil service retirement age of 60 years. These counties will likely need to make advance plans for staff recruitment even just to maintain existing nurse numbers, as well as to avoid further shortages. Also the results generally reflect that there are larger proportions of younger nurses in hardship-linked counties than in other counties. However in all counties efforts to increase the recruitment and retention of younger nurses would appear to be needed as part of efforts to build a skilled, sustainable public sector nursing workforce.

Literature has reported associations between health worker density and important health system indicators in cross-country analyses [11,12,16]. We are not aware of any prior reports from a sub-Saharan African, low-income country, that have explored such associations within one country. We provide data suggesting positive, statistically significant associations of the public sector nursing densities with health spending per capita and immunization rates. These analyses, while exploratory, indicate an association between health worker density and other markers of inequity. Although perhaps not surprising, the availability of such data and the ability to track changes over time will be important in determining whether counties individually, and Kenya as a whole, are making progress in tackling inequities.

Of note is that the association between health worker density and mortality usually may reach a certain ‘inflection’ where the addition of more workers does not reduce mortality. However it seems unlikely that nurse densities have reached such an inflection point in countries such as Kenya given the low density of health workers observed. Unfortunately lack of accurate county-specific mortality rates make examining this hypothesis impossible at present. Literature does suggest that in Kenya, maternal mortality as a health outcome can be affected by access to medical facility/distance to nearest health facility and household income [41], and that the proportion of gross domestic product spent on health, and female literacy affect health outcomes related to infant and under-five mortality rates (per 1,000 live births) [42].

Our study has some limitations. Though the availability of the Kenya Health Workforce Information System is a major step forward in supporting health workforce management within the Ministries of Health, and it is the most advanced of any health cadre-specific database in this country as well as one of the most promising systems among low-income countries, various limitations remain. For example, some inadequacies of reporting exist at facility, district, and provincial levels. However we are confident that the overall pattern of our findings is a reasonable representation of the current situation within the public sector.

Second, it is clear that mechanisms are needed which will enable information to be captured from all sectors including the faith-based, non-governmental, and private sector. Thus the information system should be supported to provide a comprehensive picture of the nursing workforce (and other cadres) in the entire country. Third, the absence of similar nursing deployment data from earlier periods makes it impossible to determine whether the picture of the nursing workforce in the public sector is generally stable, improving, or deteriorating as a result of recruitment and deployment policies. Continued investment in such information resources would make such analyses possible. The effect of the Emergency Hire Plan on the country’s health-care system has however previously been examined using the information system, and it has been documented that the program had significant impact in increasing the health services capacity in Kenya’s rural and underserved areas over the short-term [19].

We acknowledge that the health spending per capita indicator, presented publicly by the government [43], took into account only two items, based on a national household survey, as a proxy for health spending. Ideally health spending per capita should be based on the country’s total health expenditure which includes health expenditure from households, public and private sectors disaggregated by county.

Interpreting immunization rates and public nursing workforce density relationships should also be done cautiously. Immunizations can be offered in both public and private health facilities as part of the national immunization program. However the private for-profit facilities in many rural areas provide mainly curative health services with the public sector providing by far the majority of immunization services nation-wide. We acknowledge, however, that this is a limitation of such analyses and in part our report is to highlight why we should invest in more comprehensive workforce data.

## Conclusions

Human resources for health information systems are necessary for generating, managing, and disseminating knowledge on the health workforce, and provide a platform for decision making by health-care managers, local and national policymakers, and global organizations [1]. Our analyses based on the Kenya Health Workforce Information System indicates that there is an overall shortage of nurses (range of 1.2 to 0.08 per 1,000) in the public sector countrywide, and that there are major variations across counties in the nursing densities, skill mix, and demographic characteristics of the nurses. Counties may face quite different challenges related to addressing their specific nursing shortages, upgrading the training status of the nurses available to them or the imminent retirement of a substantial proportion of their workforce. Therefore current basic analyses suggest that stakeholders should address the national and county-specific nurse shortages and mal-distribution if equitable, quality health-care delivery in the counties is to be achieved. Also continued, improved efforts should be made to collate workforce data so as to allow progress in addressing the human resources for health challenge to be monitored going forward.

## Abbreviations

AIDS: Acquired immune deficiency syndrome; HIV: Human immunodeficiency virus; US: United States.

## Competing interests

The authors declare that they have no competing interests.

## Authors’ contributions

ME, MR, and AW conceived the original idea for the study. ME, MR, AW, and PM facilitated obtaining the required funding. CR, MR, AW, and RK provided support for the primary data collection, and CR provided access to the final national nursing deployment data used. MW, JT, RK, and JO cleaned and merged the data and linked to facility codes. MW and JT performed statistical analysis, while JO and AN undertook geographical analysis. MW produced the first draft of the manuscript with support from ME, JT, and PM. All authors contributed to revisions of subsequent drafts, and read and approved the final manuscript.
